# Editorial: Self-conscious emotions and group-identification - theoretical, empirical, and normative questions

**DOI:** 10.3389/fpsyg.2022.970665

**Published:** 2022-07-26

**Authors:** Alessandro Salice, Mikko Salmela, Alba Montes Sánchez, Gavin Brent Sullivan

**Affiliations:** ^1^University College Cork, Cork, Ireland; ^2^University of Copenhagen, Copenhagen, Denmark; ^3^University of Helsinki, Helsinki, Finland; ^4^International Psychoanalytic University Berlin, Berlin, Germany

**Keywords:** group identification, group-based emotions, social self, self-conscious emotions, pride, envy, shame, empathy

For the past few decades, psychological, sociological, and philosophical investigations have converged in showing that self-conscious or self-evaluative emotions like shame, pride, guilt, envy, etc. play a crucial role in building our sense of self as individuals. Importantly, these emotions have also been described as social and relational because other people may have an effect on how we feel about various dimensions of our selves. This is the case, for example, when we feel ashamed (and thus faulty, deficient, or unworthy in the eyes of others) because of shameful actions performed by other people, or when we feel pride (and thus empowered, self-assertive, or self-confident) because of others' commendable actions. Similar considerations hold for envy, where the belongings of others induce the sense of inferiority in us, which often times lead to envy.

Self-conscious emotions that display this specific social form have been labeled “hetero-induced,” “vicarious,” “reflected” (i.e., reflected glory or failure) or “group-based” (i.e., embodying or enacting self-relevant changes in group status or power). It has been suggested that an important precondition of these emotions is for their subject to undergo group-identification. Group-identification is the psychological process whereby a subject acquires a social self or a social identity (that is, an understanding of oneself as group member). On this account, only the actions, opinions or belongings of relevant others, i.e., of perceived group members have the power to impact our sense of self in self-conscious emotions. Frontiers' Research Topic on “Self-Conscious Emotions and Group-Identification—Theoretical, Empirical, and Normative Questions” explores this research hypothesis by collecting five cross-disciplinary investigations into the relations between group identification and self-conscious emotions. Each contribution illuminates the many ways in which our social identity shapes (and is shaped by) the way we feel esp. shame, pride, envy, and empathy.

In “*Shamed If You Do, Shamed If You Do Not: Group-Based Moral Emotions, Accountability, and Tolerance of Enemy Collateral Casualties*,” Schori-Eyal et al. study how perceived accountability and forecast group-based emotions contribute to caution in decision-making, thereby reducing outgroup civilian casualties in intergroup conflicts. In two studies, Jewish-Israeli civilians (Study 1) and soldiers (Study 2) were requested to forecast their group-based moral emotions regarding Palestinian civilian casualties, and then exposed to accountability manipulations. Participants who expected to feel mild shame and were primed with accountability made more cautious decisions than those in the control group. Participants who expected to feel intense shame were uninfluenced by accountability priming.

“*The Rational Appropriateness of Group-Based Pride*” by Salmela and Sullivan analyses the appropriateness of group-based pride, distinguishing between the shape and size of this emotion. For the appropriate shape of group-based pride, the authors suggest two criteria: the distinction between group-based pride and group-based hubris, and between we-mode and I-mode sociality. While group-based hubris is categorically inappropriate, both we-mode and I-mode group-based pride are appropriate if the members have collectively contributed to a group achievement. Regarding the size of group-based pride, we-mode group members are warranted to experience and express more intense pride than I-mode group members. Moreover, the proper intensity depends on whom the expression is directed toward.

In “*Influence of Group Identification on Malicious and Benign Envy: A Cross-Sectional Developmental Study*” Gaviria et al. assess the effects of group-identification on envy responses in a sample of Spanish schoolchildren. The study shows that activating a social self has a clear impact on upward social comparison and its effects. More concretely, in intergroup situations, group identification increases malicious envy toward outgroup members and significantly decreases it toward ingroup members (probably transforming it into benign envy, which is still reported toward ingroup members).

Miyazono and Inarimori in their “*Empathy, Altruism, and Group Identification*” investigate empathy-induced helping behavior. They explore how empathy (conceived of as an other-oriented emotion) can motivate helping behavior by arguing that this emotion presupposes group identification. The main claim is that, when X empathizes with Y and, as a consequence, helps Y, they do so because they understand Y as a member of their group and they, thereby, understand Y's welfare as constitutive of their in-group's welfare. Empathy-induced helping behavior therefore escapes the traditional dichotomy of egoism vs altruism insofar as help can be described as altruistic at individual level, but egoist at group level.

In their article, “*I Feel Different, but in Every Case I Feel Proud: Distinguishing Self-Pride, Group-Pride, and Vicarious-Pride*,” De Hooge and van Osch present a series of experiments which show that different feelings are associated with three forms of pride about three different “objects”—which correspond to (1) being proud of myself, (2) proud of the group in which I am included and partially responsible for an achievement (e.g., an academic or professional team), or (3) feeling proud of someone else. Differentiation of these three forms of pride is empirically demonstrated to be related to senses or feelings of: being responsible and “self-inflated” in the case of self-pride, admiration of and more positive feelings toward a specific other person in the case of vicarious-pride, and closeness to others in the case of group-pride (i.e., to the other members of one's team). Future research should explore how the different forms of pride have different behavioral outcomes and may vary due to further individualistic and collectivistic cultural factors.

All in all, these articles paint a detailed picture of the various ways in which our social affiliations impact and modulate our self-conscious emotions and, thereby, our sense of self.

## Author contributions

All authors listed have made a substantial, direct, and intellectual contribution to the work and approved it for publication.

## Funding

AM wishes to acknowledge that her work on this Research Topic has been funded by the European Union's Horizon 2020 research and innovation programme under the Marie Skłodowska-Curie Grant Agreement No. 890316.

## Conflict of interest

The authors declare that the research was conducted in the absence of any commercial or financial relationships that could be construed as a potential conflict of interest.

## Publisher's note

All claims expressed in this article are solely those of the authors and do not necessarily represent those of their affiliated organizations, or those of the publisher, the editors and the reviewers. Any product that may be evaluated in this article, or claim that may be made by its manufacturer, is not guaranteed or endorsed by the publisher.

